# Spatial analysis of HIV-TB co-clustering in Uganda

**DOI:** 10.1186/s12879-019-4246-2

**Published:** 2019-07-12

**Authors:** Augustus Aturinde, Mahdi Farnaghi, Petter Pilesjö, Ali Mansourian

**Affiliations:** 1 GIS Centre, Department of Physical Geography and Ecosystem Science, Lund University, SE-221 00 Lund, Sweden; 2 College of Computing and Information Science, Makerere University, Kampala, Uganda; 3 Department of Lands and Architectural Studies, Kyambogo University, Kampala, Uganda; 4 Centre for Middle Eastern Studies, Lund University, Sölvegatan 10, 223 62 Lund, Sweden

**Keywords:** HIV, TB, TB/HIV co-infection, Spatial co-clustering, Spatial scan statistics, Moran’s I, Bivariate Moran’s I, Uganda

## Abstract

**Background:**

Tuberculosis (TB) is the leading cause of death for individuals infected with Human immunodeficiency virus (HIV). Conversely, HIV is the most important risk factor in the progression of TB from the latent to the active status. In order to manage this double epidemic situation, an integrated approach that includes HIV management in TB patients was proposed by the World Health Organization and was implemented in Uganda (one of the countries endemic with both diseases). To enable targeted intervention using the integrated approach, areas with high disease prevalence rates for TB and HIV need to be identified first. However, there is no such study in Uganda, addressing the joint spatial patterns of these two diseases.

**Methods:**

This study uses global Moran’s index, spatial scan statistics and bivariate global and local Moran’s indices to investigate the geographical clustering patterns of both diseases, as individuals and as combined. The data used are TB and HIV case data for 2015, 2016 and 2017 obtained from the District Health Information Software 2 system, housed and maintained by the Ministry of Health, Uganda.

**Results:**

Results from this analysis show that while TB and HIV diseases are highly correlated (55–76%), they exhibit relatively different spatial clustering patterns across Uganda. The joint TB/HIV prevalence shows consistent hotspot clusters around districts surrounding Lake Victoria as well as northern Uganda. These two clusters could be linked to the presence of high HIV prevalence among the fishing communities of Lake Victoria and the presence of refugees and internally displaced people camps, respectively. The consistent cold spot observed in eastern Uganda and around Kasese could be explained by low HIV prevalence in communities with circumcision tradition.

**Conclusions:**

This study makes a significant contribution to TB/HIV public health bodies around Uganda by identifying areas with high joint disease burden, in the light of TB/HIV co-infection. It, thus, provides a valuable starting point for an informed and targeted intervention, as a positive step towards a TB and HIV-AIDS free community.

## Introduction

Tuberculosis (TB) is an airborne bacterial disease caused by *Mycobacterium tuberculosis* that most often affects the lungs. The World Health Organization (WHO) has estimated that about 10.4 million people fell ill with TB, and 1.7 million died from the disease in 2017 [1]. Human immunodeficiency virus (HIV) is one of the most important risk factors responsible for the progression of latent TB to active TB [2]. People living with HIV have a 20-fold higher risk of developing TB than those without HIV, and the risk continues to increase as the vital immunity cells (CD4) count progressively decreases [3]. HIV/TB co-infection is thus known as a ‘double trouble’ [4] and a public health threat especially for regions where both diseases are endemic.

Sub-Saharan Africa carries the biggest burden of both diseases, with 95% of global TB deaths and more than 70% of the global HIV burden [5]. Uganda, like the rest of Sub-Saharan countries, is plagued by the dual TB and HIV epidemics and is the seventh in the 22 countries with the highest TB prevalence [1]. Whereas Uganda’s HIV prevalence has reduced to 6.0% in 2016 (from 7.3% in 2011, among 15–49 years old), it was still estimated that 1.3 million individuals were infected with HIV [6]. With TB/HIV co-infection at 41.5%, TB is the leading preventable cause of death among people with HIV, responsible for over 30% of HIV deaths [7].

To decrease the combined TB/HIV prevalence, the WHO formulated a framework in 2005 (modified in 2012), that aims at collaborating TB/HIV activities to manage TB in HIV patients [8]. This position is re-echoed by the WHO in its strategy to end TB in the post-2015 era of the Sustainable Development Goals (SDG) [9, 10]. The motivation for simultaneous management of TB/HIV was largely informed by the proven interactions between TB medication and HIV medication, leading to the ineffectiveness of the TB medication [3]. Additionally, both diseases complement each other with HIV quickening the progression of TB, and vice versa, for co-infected patients [11].

Due to the importance of TB and HIV co-infection, a number of scholars have endeavored to study the correlation between the two diseases. For example, while studying HIV and TB prevalence in New York, Wallace et al. [12] observed that whenever HIV infection was high in the population, there were also high numbers of patients with tuberculosis. Additionally, Corbett et al. [13], having used global TB and HIV prevalence data, concluded that both diseases exhibited similar patterns in both space and time. From a geographical perspective, Wei et al. [14] observed similar spatial clustering patterns between TB and TB/HIV co-infection in Xinjiang province, China. Similarly, Ross et al. [15] used bivariate choropleth mapping and showed that both TB and HIV were correlated and that the joint distribution for both diseases was spatially heterogeneous across Brazil. Their outputs provided an information basis for targeted intervention by the public healthcare bodies responsible for TB and HIV.

However, due to the historical lack of geographically referenced disease records, as well as lack of reliable statistics on morbidity and mortality in most African countries with high TB/HIV disease burden [6, 16], few studies have considered the simultaneous spatial patterns of these comorbidities in Africa. Luckily, with the introduction of District Health Information Software 2 (DHIS2), an open source software platform developed by Health Information System Program (HISP) to African countries, healthcare admission data for most diseases can now be recorded, hierarchically, from local to national levels [17]. For example, Gwitira et al. [5] used DHIS2 data from Zimbabwe to investigate the spatial overlaps in the distribution of HIV/AIDS and malaria. They identified 5 out of the 71 districts as clusters having high records for both HIV and malaria. These would be areas where efforts targeting minimizing both diseases would pay special attention.

In line with the WHO recommendation for collaborative management of TB and HIV, we argue that it is logical to establish the spatial joint distribution of these two co-infections in order to inform local and national intervention strategies. Whereas some studies have examined the individual spatial clustering of TB and HIV both elsewhere [18–21] and in Uganda [22], an intervention based on only one of the two complementary diseases would be ineffective.

Given that the spatial perspectives of HIV/TB co-infection are yet to be studied in Uganda, our main aim of this study, therefore, is to examine the spatial clustering of TB and HIV prevalence rates in Uganda for a three-year period (2015 to 2017) – with particular emphasis on spatial co-clustering. To the best of our knowledge, this is the first spatial study to consider co-clustering of both diseases at a national scale in Uganda. We use spatial-clustering detection and analysis techniques to identify significantly persistent clusters for TB and HIV, providing an informed basis to the Ministry of Health and partners, on the location of such co-clusters thereby potentially aiding effective joint TB/HIV intervention.

## Methods

### Study area

The study is carried out in Uganda, a country located within East Africa, and about 800 km from the Indian Ocean. Uganda is landlocked bordered by Kenya in the East, South Sudan in the North, Democratic Republic of Congo in the West, Tanzania in the South, and Rwanda in South West. It has a total area of 241,551 km^2^, of which the land area covers 200,523 km^2^. Administratively, the country is divided into one city and 122 districts (as of 2018) that are further subdivided into counties, sub-counties, parishes, and villages. Uganda’s climate is equatorial with the mean temperature range of 16 °C to 30 °C, even when the Northern and Eastern regions sometimes experience relatively high temperatures exceeding 30 °C and the South Western region sometimes has temperatures below 16 °C. The relief of the study area ranges from 614 m (above mean sea level) to 5,111 m at the highest point. The 2014 national census estimated the population of Uganda to be about 35 million people.

### Data

TB and HIV admission records were obtained from the DHIS2 system that is housed by the Ministry of Health of Uganda. The DHIS2 system is a community-based aggregation health information system that scales from the lowest level to the national level [23]. The annual TB and HIV were recorded at the geocoded government healthcare facilities distributed throughout the country and aggregated to the district level. The recorded TB and HIV were all diagnosed cases, for patients that tested at centers located within a specific district. Records from 2015 to 2018 were obtained. However, at the time of acquisition (June 2018), only half of 2018 were recorded and therefore the 2018 records were excluded from the analysis.

Whereas HIV-TB coinfection records were retrievable from the DHIS2 system, they were deemed unreliable (by the staff) mainly because many health units that report to the DHIS2 do not have the capability of diagnosing both HIV and TB simultaneously. They thus report HIV and TB separately. In total, TB and HIV records were obtained for 122 districts in Uganda (based on 2018 administrative boundaries). District level population data and the district mapping shapefiles were obtained from Uganda Bureau of Statistics (https://www.ubos.org/).

**Fig. 1:**
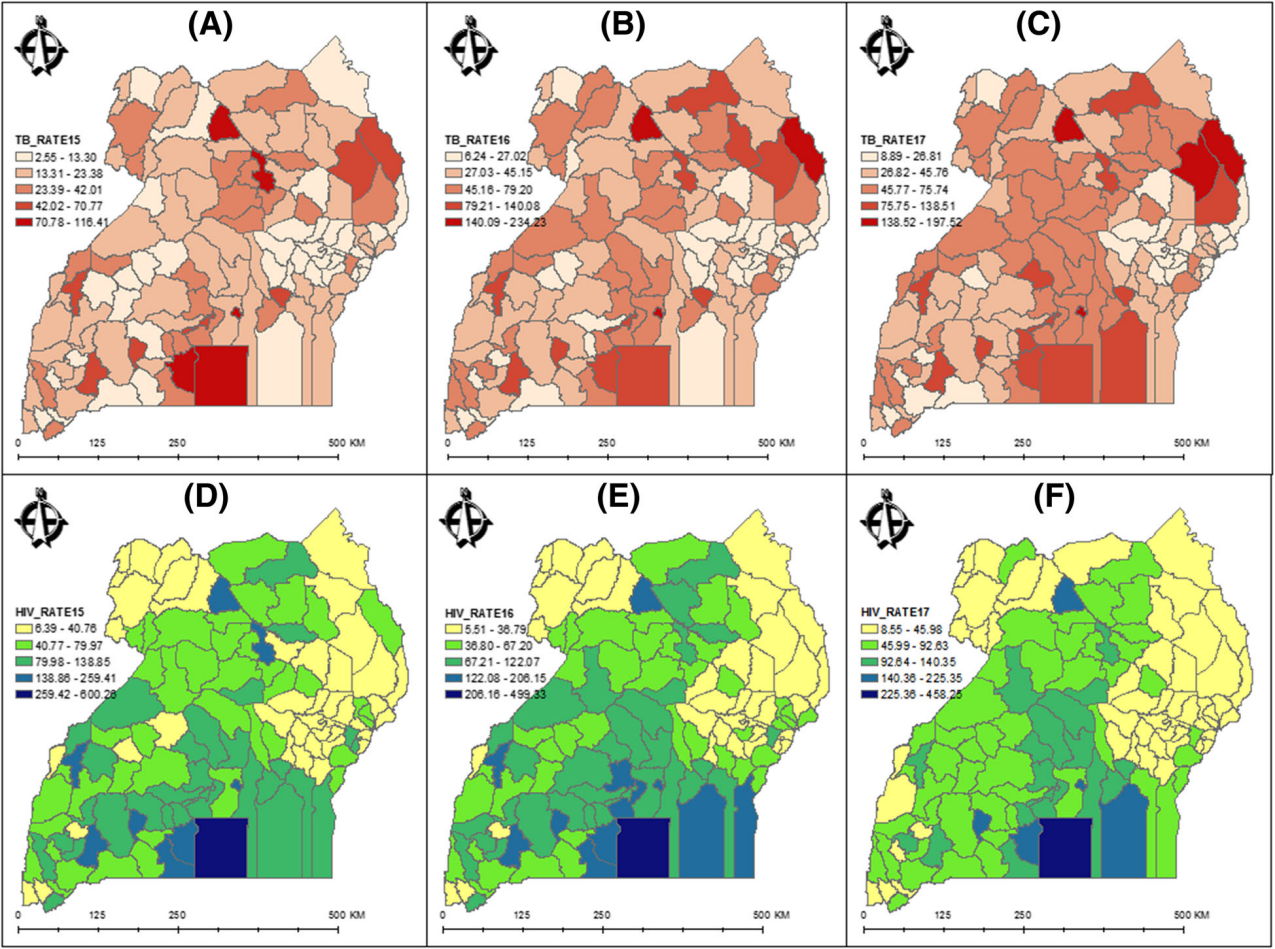
TB and HIV prevalence rates per 10,000 people in Uganda from 2015 to 2017 (**a**, **b** and **c** for TB; **d**, **e** and **f** for HIV)

TB and HIV admission counts were spatially joined to their respective district polygons for 2015, 2016 and 2017. The TB and HIV disease prevalence was calculated by dividing the total number of each disease cases in each district by the total human population in the district to obtain the population-adjusted district level prevalence rates. For all the years, the population used was that from the 2014 Uganda national census, and the resultant trends are visualized through Fig. 1. As can be observed, the prevalence rates for both TB and HIV, for any given year, are not uniform across Uganda.

### Statistical analysis

To understand the characteristics of the TB and HIV data, global pattern analysis was conducted. This involved computing for Spearman’s correlation – an overall measure of the linear relationship between TB and HIV district-recorded prevalence rates. The influence of neighborhood prevalence rates on the district-observed prevalence rates was also investigated. This spatial tendency is known as spatial autocorrelation and was globally investigated by using global Moran’s Index and bivariate global Moran’s Index that identified whether the data were spatially autocorrelated or not. Then, spatial scan statistics (SaTScan) was used to extract the local spatial clusters and mark the areas of high risk to inform prevention intervention.

The global analyses were concerned with summarizing the trends within the data, when viewed at a Uganda national level, for the years 2015, 2016 and 2017. SaTScan was concerned with identifying the location and the shape of significant local clusters (hotspots and cold spots) in the study area.

Given that reliable records for HIV-TB coinfection were not available (as discussed in section 2.2), bivariate local Moran’s Index was used to investigate the simultaneous occurrence and hence co-clustering in both TB and HIV. It reports areas with hotspots (High-High), cold spots (Low-Low) and discordant (High-Low or Low-High) clusters. To ensure the robustness of the obtained clusters, 9,999 randomizations were allowed for this analysis.

#### Global pattern analysis

Spearman’s correlation analysis was used as a statistical measure for the strength of the linear relationship between TB and HIV district-paired data. The global Moran’s I was used to examine the spatial auto-correlation in the TB and HIV prevalence rates. Generally, spatial autocorrelation can be understood as the measure of the influence that the neighborhood values have on the observed values [24–26]. It stems from Tobler’s first law of Geography: “everything is related to everything else, but near things are more related than distant things” [27]. This required computation of contiguity information through the generation of the spatial weight matrix. Rooks contiguity was used in this study [28]. Moran’s I relates the average TB or HIV prevalence rate within each neighborhood (spatial lag) and the standardized TB or HIV prevalence rate. The global Moran’s I and bivariate global Moran’s I were performed using GeoDa software [29].

#### Spatial scan statistics

District-specific TB and HIV clusters were detected by applying Kulldorff’s spatial scan statistics [30]. The same technique has been widely used in many applications [14, 18–20, 22, 31]. Spatial scan statistics have reasonable sensitivity and specificity [32]. This enhances their efficiency and accuracy when compared with other cluster detection methods, such as Bayesian disease mapping [5]. The Spatial clusters were detected based on the Poisson probability model, with the underlying assumption that the observed TB and HIV cases in each district, when adjusted for the population at risk, result from a random process [32].

The basic idea behind SaTScan is to impose circular windows of various sizes across the study area, and at each location, defined by the district centroid location in this study; a comparison is made between the disease rate within the window and that outside of it. Under the discrete Poisson assumption, SaTScan [33] detects potential clusters by calculating the likelihood ratio (LR) given by eq. (1).

1 $$ {LR}_{(u)}={\left(\frac{c}{E_{\left[c\right]}}\right)}^c{\left(\frac{C-c}{C-{E}_{\left[c\right]}}\right)}^{C-c}I\left(\frac{c}{E_{\left[c\right]}}>\frac{C-c}{C-{E}_{\left[c\right]}}\right) $$

where *C* is the total number of TB or HIV cases in the study area; *c* is the observed number of TB or HIV cases within a circle; *E*_[*c*]_ is the adjusted expected number within the window under the null hypothesis; *C* − *E*_[*c*]_ is the expected number of TB or HIV cases outside the window, and $I\left(\frac{c}{E_{\left[c\right]}}>\frac{C-c}{C-{E}_{\left[c\right]}}\right)$ is the binary indicator of high-risk clusters (1) or low-risk clusters (0) or both (11). Based on the magnitude of the values of the likelihood ratio test, the set of potential clusters are then ranked and ordered. The circle with the maximum likelihood ratio among all radius sizes at all possible centroid locations is considered as the most likely cluster. The statistical significance of the clusters is determined through Monte Carlo simulations (999 simulations).

Within the SaTScan software, the “spatial” option to 2015, 2016, and 2017 TB and HIV case data, both High and Low rates (Hotspots and Coldspots) were analyzed. The user-defined maximum radius of the circular spatial window was varied, starting at 5% and incremented by 5% until it reached 50%. The obtained results were not affected by the choice of the radius selected. The default value of 50% of the population at risk, as advised by Kulldorff [30] was thus maintained.

#### Bivariate local Moran’s I

The bivariate local Moran’s I, also called BiLISA, is an extension of the univariate local Moran’s I to model the correlation between one variable (e.g. TB) at a location, and a different variable (e.g. HIV) at the neighboring locations. The bivariate Moran’s I (for TB) of the *i* th district can be calculated as eq. (2).

2 $$ {I}_i=\frac{\left({x}_{TB}-{\overline{x}}_{HIV}\right)\sum \limits_j{w}_{ij}\left({x}_{TB}-{\overline{x}}_{HIV}\right)}{S^2} $$

where *x*_*i*_ = the TB prevalence rate for the *i* th district; $\overline{x}$ = the mean HIV prevalence rate for all districts in the study area; *x*_*j*_ = the TB prevalence rate for the *j* th district; *w*_*ij*_ = a weight parameter for the pair of districts *i* and *j* that represent proximity; S = the standard deviation of the TB prevalence rates in the entire study area. The same was done for HIV, with TB and HIV switching positions.

## Results

### Global pattern analysis

Table 1 represents these global summary statistics for the study period.

**Table 1:** Moran’s I and Correlation for TB and HIV (2015–2017)

	2015	2016	2017
Moran’s I			
TB	0.118	0.069	0.129
HIV	1.239	0.327	0.377
Bivariate Global Moran’s I			
TB/HIV	0.112	0.074	0.110
Spearman’s Correlation			
TB/HIV	0.759	0.548	0.602

It can be observed that for both diseases, the Moran’s I is significantly positive (at 95% confidence interval) – disqualifying the null hypothesis that observations are spatially independent (Moran’s I of zero). The positive Moran’s I values in Table 1 show that neighboring districts tend to have similar prevalence rates for both TB and HIV. Also, for the whole study period, HIV was consistently more spatially correlated than TB. The significantly positive bivariate Moran’s I showed that overall, the observed TB rates were positively influenced by the HIV rates in the neighborhood and vice versa. The computed Spearman’s correlation showed that the two diseases were highly correlated through the correlation varied with time. The correlation was highest for 2015 (76%), lowest for 2016 (55%) and moderately high (60%) for 2017.

### Clustering analysis

**Fig. 2:**
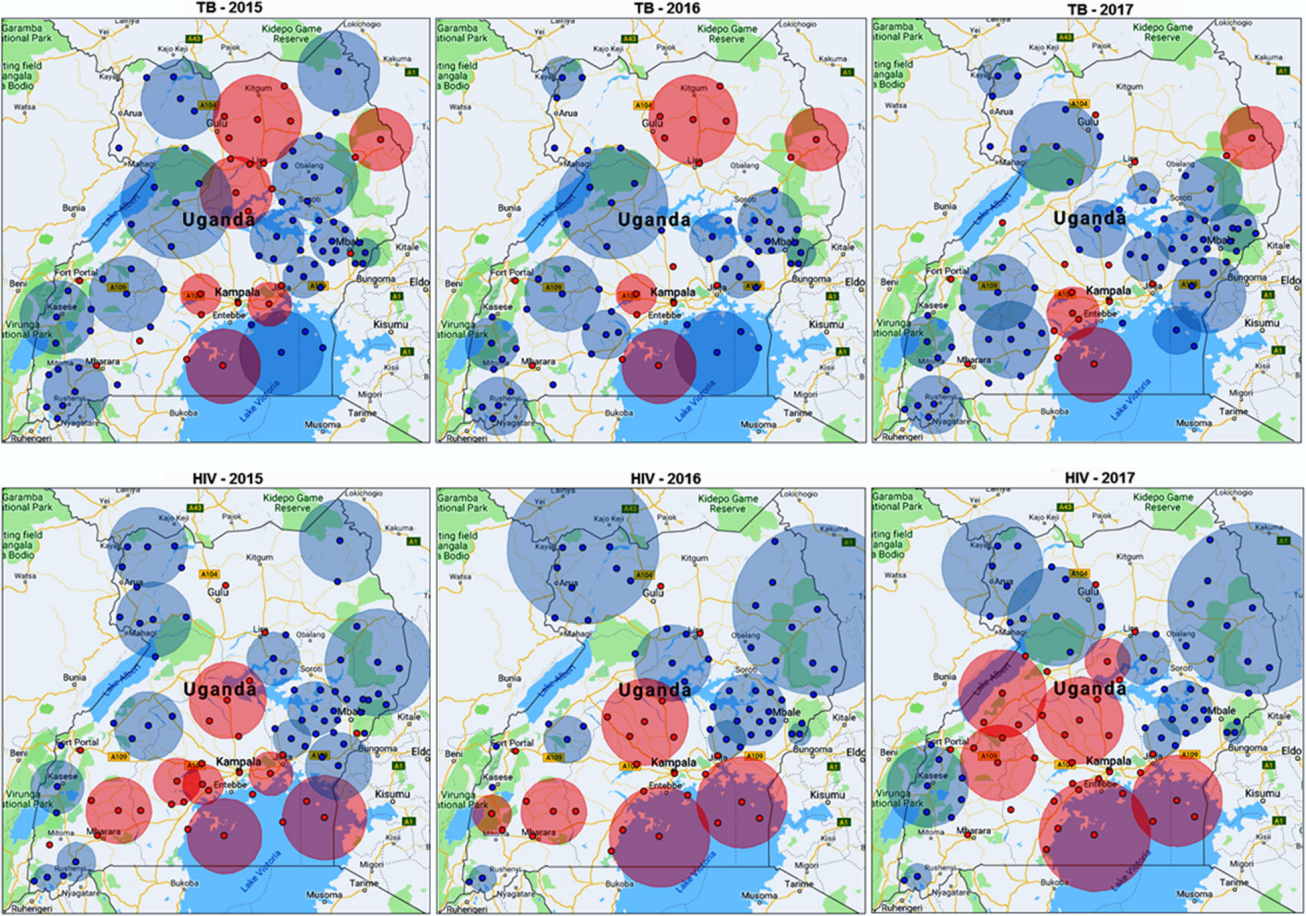
TB and HIV High (RED) and Low (BLUE) clusters across Uganda (2015–2017)

To distill out areas with probable clusters of TB and HIV, spatial scan statistics (discrete Poisson) were employed and the result is shown in Fig. 2.

It can be observed in Fig. 2 that TB high clusters were largely around Lake Victoria and in the central north and one consistent high cluster in the northeast. There is a noticeable reduction in the number of big high clusters from 2015 (six), to 2016 (four), and 2017 (three). The TB low clusters were concentrated in the West and the East (with the central axis dominated with high clusters). On the other hand, HIV high clusters were consistently concentrated in the south, around Lake Victoria and the central parts of Uganda, throughout the study period. The low clusters were generally concentrated in the east, northeast, northwest, and southwest.

### Co-clustering analysis

**Fig. 3:**
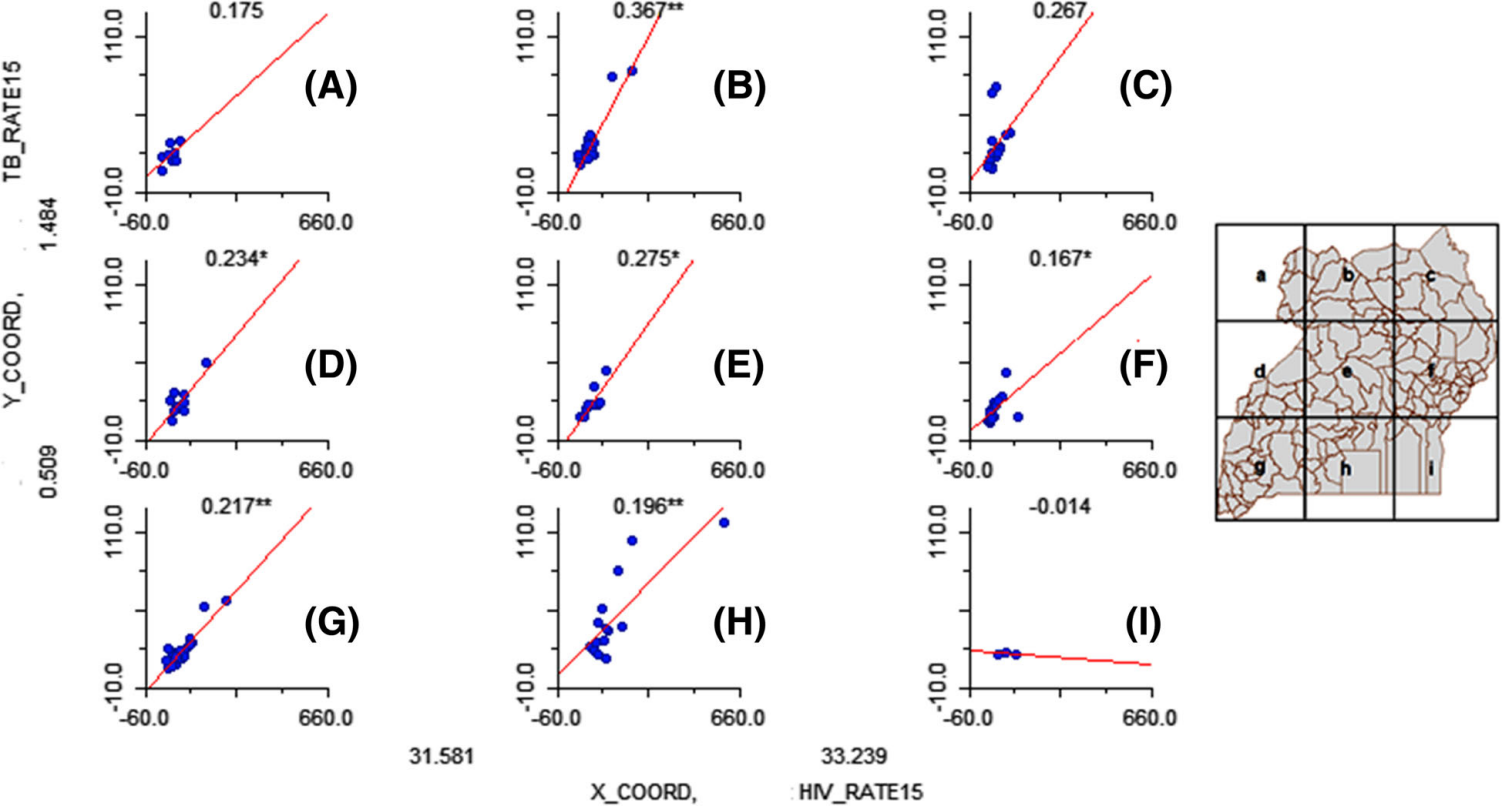
Spatial variation of TB condition on HIV across Uganda, 2015. Letters **a**-**i** represent the regions from which the variations are derived as shown by the map on the right

To this end, the concentration has been on the spatial global trends or local clustering patterns in the individual disease prevalence rates. To investigate the simultaneous variation of TB and HIV prevalence in Uganda, the study area was segmented into 9 regions (“bins”) based on the study area coordinates, and the linear relationships between the prevalence rates regenerated. Given that relatively similar clustering trends were observed throughout the study period, it was considered that any single year would be representative of the study period. Figure 3 shows the resultant relationships after regionalization, for 2015.

Figure 3 shows the spatial variation of TB-HIV relationship across Uganda for 2015 (the pattern is observed for 2016 and 2017). It illustrates that across Uganda, TB generally had a positive association with HIV and this relationship varies significantly across the geographical space. For example, across the diagonal (plots g, e, and c), the gradient is consistently around 0.2 and significant (at 95% confidence interval) for plots g and e, and not significant for plot c. The region with the highest spatial relationship between the two diseases is the middle upper-most region (b). The lowest right region (plot i) has a negative relationship between TB and HIV, though it is not statistically significant.

**Fig. 4:**
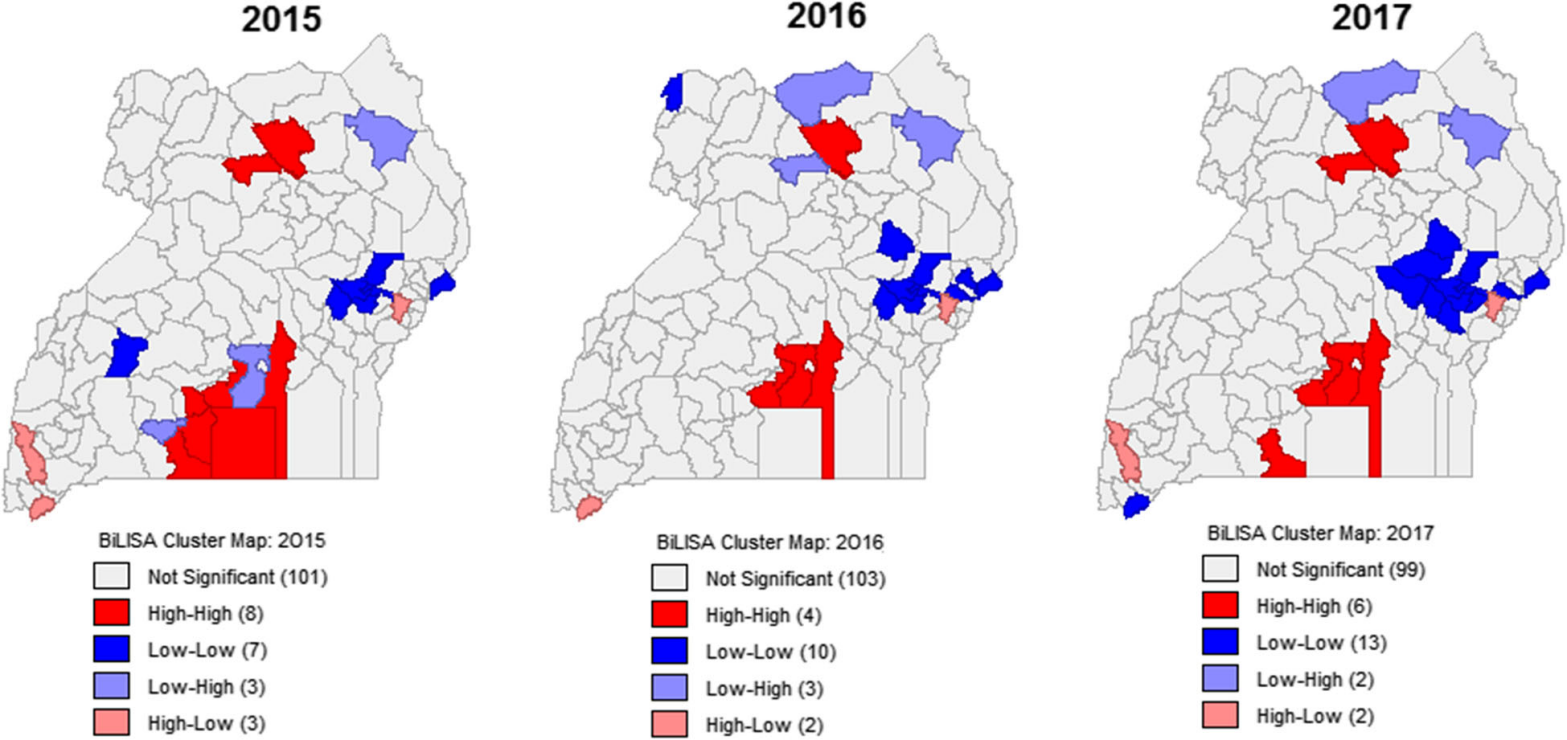
Spatial Co-clustering of TB and HIV across Uganda (2015–2017)

To model the simultaneous occurrence and hence co-clustering of both diseases in space, the bivariate local Moran’s I was used to show areas where similar disease rates were clustered; characterizing the resultant clusters into High-High, Low-Low, Low-High and High-Low clusters as shown by Fig. 4.

Figure 4 illustrates that generally, there are two High-High TB/HIV occurrence and co-clusters: one around Lake Victoria consisting districts of Kalangala, Mpigi, Kyotera, Kalungu, Masaka, and Mukono, and the other in the north-central districts of Pader and Omoro, in 2015. There is a Low-Low TB/HIV occurrence and co-cluster in the east consisting districts of Butebo, Kaliro, Pallisa, Kumi, Bukwo, and Kibuku, and another central-west co-cluster in Kyegegwa district. Six districts appear as discordant clusters with Lwengo, Wakiso, and Kotido appearing as Low-High, while Rukungiri, Kabale, and Mbale appear as High-Low, for 2015.

For 2016 and 2017, the trends in TB/HIV occurrence and co-clusters are more or less the same as for 2015 with generally a High-High TB/HIV occurrence and co-cluster around the Lake Victoria region and north-central, and a Low-Low occurrence and co-cluster in the east that progressively increase in size with time. For 2016, Koboko in the northwest appears as a cold cluster, though it again became insignificant in 2017. Apart from Mbale and Kotido discordant clusters that are consistent throughout the study period, other discordant clusters (Rukungiri, Kabale, Lamwo, Omoro) are temporally unstable. Also, the districts of Kalangala, Masaka, and Kyotera, consisting of the lower south High-High co-cluster, are unstable throughout the study period.

## Discussion

Epidemiological intervention based on the homogeneity of disease patterns often results in non-optimised utilization of the available resources where resources are dedicated to areas that do not require them, at the expense of the areas that require them more [34]. Through the use of spatial methods, health outcomes data can be distilled into their spatial heterogeneity, providing a basis for the explanation of the observed heterogeneity on the basis of existing local risk factors [35].

Our analysis shows that TB and HIV prevalence is geographically heterogeneous. This spatial variability is consistent with the results from the 2016 Uganda Population HIV Impact Assessment (UPHIA) which indicated that the magnitude of HIV prevalence varied considerably across Uganda from a low of 2.8% in West-Nile to 7.7% in the southwestern region [7]. Similarly, our results were consistent with those from the first nationwide community-based TB prevalence survey in 2014/15. Here, it was established that TB was about 1.3 times more prevalent among the urban population than rural residents; approximately three times more prevalent among men than women; nearly three times more prevalent among HIV-negative than HIV-positive individuals; and that TB hotspots exist in both urban and rural areas [36].

These two national surveys for HIV and TB confirm that both epidemics significantly vary across the Ugandan geographic space. However, they do not explicitly identify where the disease clusters are located, making targeted intervention difficult if not impossible. In our study, we identified the clusters exhibited by each disease, as well as the combined occurrence and clustering of both diseases. We also found that the two diseases were highly correlated, hence qualifying the need to manage both diseases simultaneously [9, 37]. Our analysis found a 76, 55, and 60% correlation between TB and HIV for 2015, 2016 and 2017, respectively. This was consistent with results by Dye [16] who observed up to 50% correlation between the two diseases in South Africa, Zambia, and Zimbabwe.

Even with such high correlation, TB and HIV show relatively different spatial clustering patterns across Uganda, as observed in the location of clusters in Fig. 2. For example, there were consistent TB hotspots in the greater northern and north-eastern parts of Uganda. This trend was not the same for HIV whose clusters were persistently concentrated around central and southern parts of Uganda, especially around districts in or surrounding Lake Victoria. Also, persistent cold spot clusters for both HIV and TB were observed in the eastern, north-western, and the very south-western (around Kabale) districts of Uganda. These low prevalence rates, especially for HIV, were consistent with projections by the United Nations Programme on HIV/AIDS [38].

Given that in Uganda HIV is more studied than TB, and considering the contribution of HIV in TB progression within TB/HIV co-infected persons [11], the observed TB/HIV geographical clustering trends can easily be explained from an HIV than from a TB standpoint. In Uganda, HIV was first discovered in a rural fishing community of Rakai district, in 1982 and some of the patients surveyed then had TB [39]. Since then, HIV has spread to almost all parts of the country, with some areas more affected than others, so that a more recent study by Bbosa et al. [40] found that these fishing communities are no longer sources but sinks of HIV infection. Even still, this geographical variability in HIV, which is the main risk factor for the progression of latent TB to active TB [4, 9, 14], can be explained by the variability in the underlying socio-economic, behavioral, and cultural factors [41]. Apart from HIV, other population-level risk factors for TB include poor living and working conditions, malnutrition, smoking, diabetes, alcohol abuse, poverty, contact with persons with active TB (health workers, family members), overcrowding and indoor air pollution [42–44].

The most pronounced TB/HIV hotspot co-cluster observed in this study consisted of districts around Lake Victoria; it is thus worth discussing the most likely risk factors around the lake regions. Uganda’s fishing communities have been listed among the most-at-risk population with the highest prevalence rate of 15–40% compared to 7% in the general population [36]. In an exclusive study about HIV infections in the fishing communities of Lake Victoria, Opio et al. [45] found the HIV prevalence to be 22%. They also found that these communities were underserved with HIV prevention, care, and support services when compared with other communities. Also, previous studies have shown that fishing communities have fatalistic attitudes, with some viewing HIV infection as less risky than drowning while fishing [46]. Moreover, Ondondo et al. [41], while studying the fishing communities on the Kenyan side of Lake Victoria concluded that the high HIV prevalence (23.3%) could be explained by high-risk unsafe sex practiced within fishing communities. We thus think that the TB/HIV hotspot around Lake Victoria is driven by the high HIV prevalence rates among the fishing communities, explained by confounding overlap of lack of TB/HIV support, behavioral and high-risk sex life.

This study observed another TB/HIV hotspot co-cluster in northern Uganda (Pader and Omoro). Its presence could be attributed to the presence of refugees, mainly from South Sudan, and to people that were initially internally displaced into camps, during the Lord’s Resistance Army (LRA) war that happened in northern Uganda until 2008. Refugee camps and congested places have been shown to increase TB prevalence [6] and the HIV/AIDS is also known to progress in such settings [47] even when this complex relationship is not well documented [48]. What is not contested, however, is that living in such camps reduces the communities’ resilience to such epidemics [48].

The contribution of HIV to the observed TB/HIV co-clustering notwithstanding, one cannot rule out the contribution of other known TB risk factors. These factors were discussed by Narasimhan et al. [49] and were characterized into personal factors, including age, gender, proximity to active TB, malnutrition, diabetes, and environmental factors, including overcrowding, smoking, occupational risk, dangerous alcohol consumption, indoor air pollution.

Finally, this study observed consistent TB/HIV cold spots, especially in eastern Uganda. These were areas, around Mbale district (discordant cluster), that had low prevalence rates for both TB and HIV – consistent with district estimates by UNAIDS [38], especially for HIV. This eastern cold spot could be linked to the traditional practice of male circumcision among the people in those communities – Bagisu and Sebei [50]. Also, from the HIV clusters observed in Fig. 2, Kasese district (inhabited mainly by Bakonjo) has a consistent cold cluster. Male circumcision has for long been associated with reduced risk in acquiring HIV infection. The World Health Organization, based on male circumcision studies from Kisumu in Kenya [51], Rakai district in Uganda [52], and an earlier study from South Africa [53] that had realised 53, 51, and 60% reduction in HIV acquisition risk, respectively, recommended safe male circumcision as an additional measure to reduce HIV acquisition in men [54]. Relatedly, Opio et al. [45] observed higher prevalence rates in uncircumcised men (27%) compared to their circumcised counterparts (11%). We thus submit that the observed TB/HIV cold spot clusters could be attributed mainly to low HIV prevalence rates, which are in turn mediated through culturally practiced male circumcision practices.

Whereas this study achieved its set objective of analyzing the areas in Uganda with elevated prevalence rates for HIV and TB, there were some limitations, especially regarding data availability. Data were available at the district level – which is a larger aggregate level. These results could be more informative had the analysis been done on a finer geographical level (like parish or village level). Also, data about other risk factors for both TB and HIV was not available – this data would have been used to do a more informative spatial regression analysis. These aspects shall be considered in future studies.

## Conclusions

Given that for most HIV patients, TB is responsible for more than half the mortalities, and given that HIV increases the chances of developing active TB by up to 20-folds, scientific evaluation of places where these two diseases are persistently prevalent is not only important but essential for effective management of both diseases. Our study analyzed for joint spatial clustering of TB and HIV. To the best of our knowledge, this is the first spatial study to consider both diseases at a national scale in Uganda, using DHIS2 data. By identifying areas where both diseases co-cluster for the period 2015 to 2017, this study provides valuable information to healthcare policy concerned with these two complementary and endemic diseases in Uganda.

Our analysis identifies the middle-south regions around Lake Victoria (Kalangala, Masaka, Rakai, Mukono, Wakiso, and Mpigi) and some districts in northern Uganda (Pader and Omoro) to be of special interest, as they constitute hotspots. The districts of Kabale and Mbale constitute discordant districts (areas of relatively high prevalence rates in the neighborhood of low prevalence rates, and vice versa) while other eastern districts are significantly cold spots. By aligning healthcare policy and intervention efforts with this obtained spatial heterogeneity in both disease prevalence rates, our study provides an informed starting point towards simultaneous management of TB and HIV.

## Data Availability

The datasets used and/or analyzed during the current study are available from the corresponding author on reasonable request.

## Abbreviations

DHIS2: District Health Information Software 2
HISP: Health Information System Program
HIV: Human immunodeficiency virus
LRA: Lord’s Resistance Army
SDG: Sustainable Development Goals
TB: Tuberculosis
WHO: World Health Organization
